# Supplementary figures and images for: Alterations of Phenylpropanoid Biosynthesis Lead to the Natural Formation of Pinkish-Skinned and White-Fleshed Strawberry (Fragaria × ananassa)
Source: Int J Mol Sci. 2022 Jul 1;23(13):7375. doi: 10.3390/ijms23137375 (PMC9267004; doi:10.3390/ijms23137375)

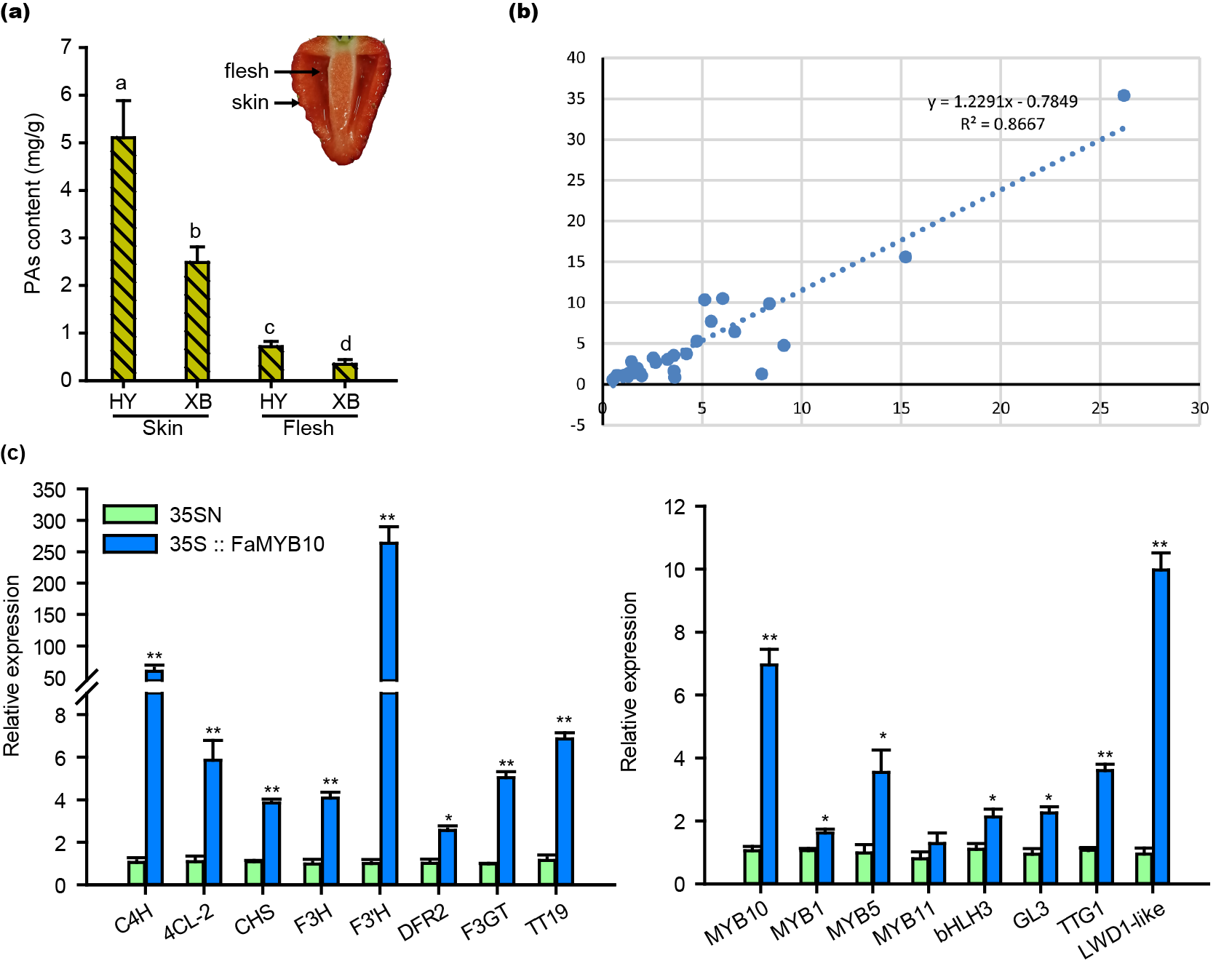

Supplement: Supplementary file 1 [file ijms-23-07375-s001.zip › Supplemental Figures/Figure S1.tif]

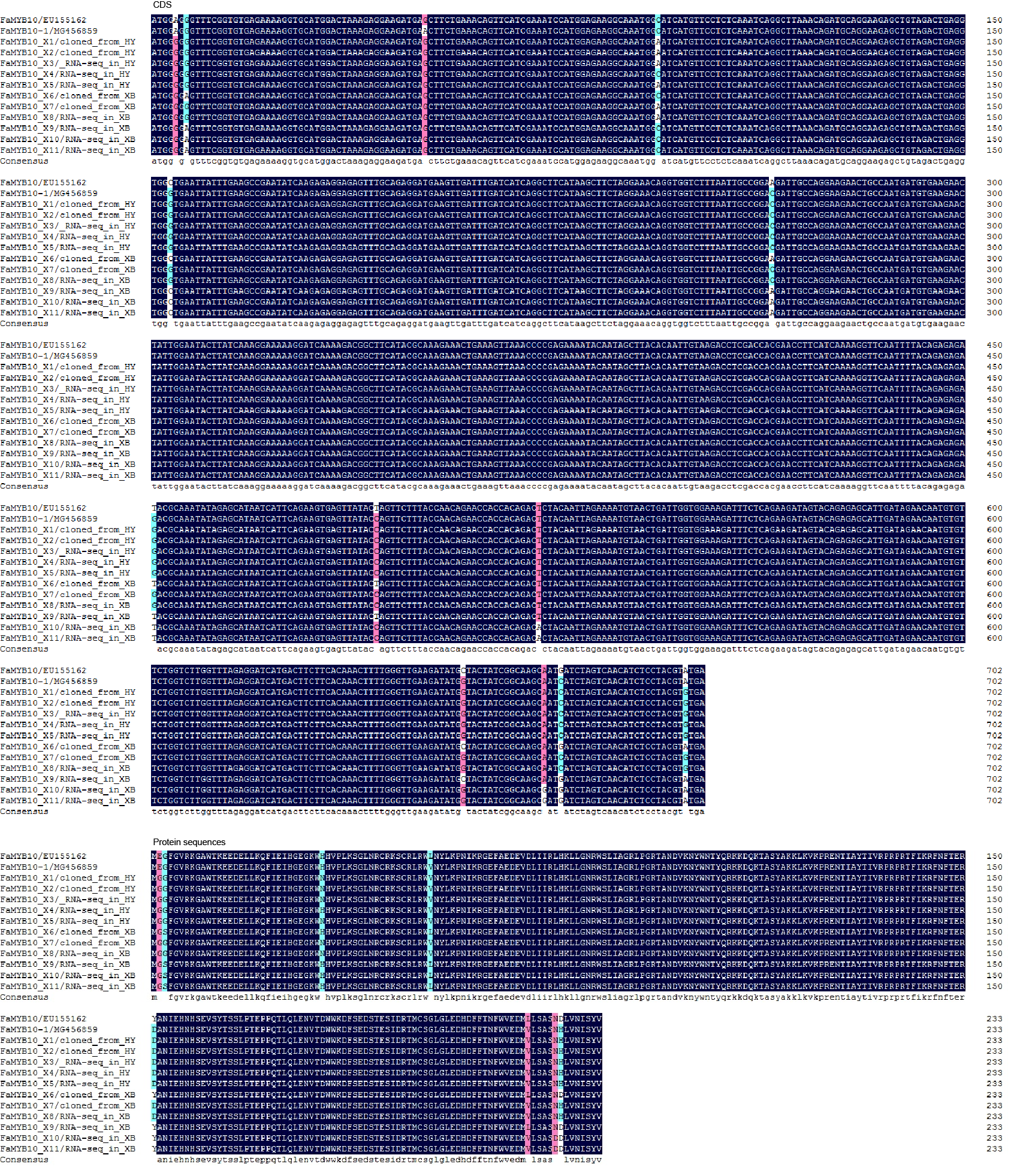

Supplement: Supplementary file 1 [file ijms-23-07375-s001.zip › Supplemental Figures/Figure S2.tif]

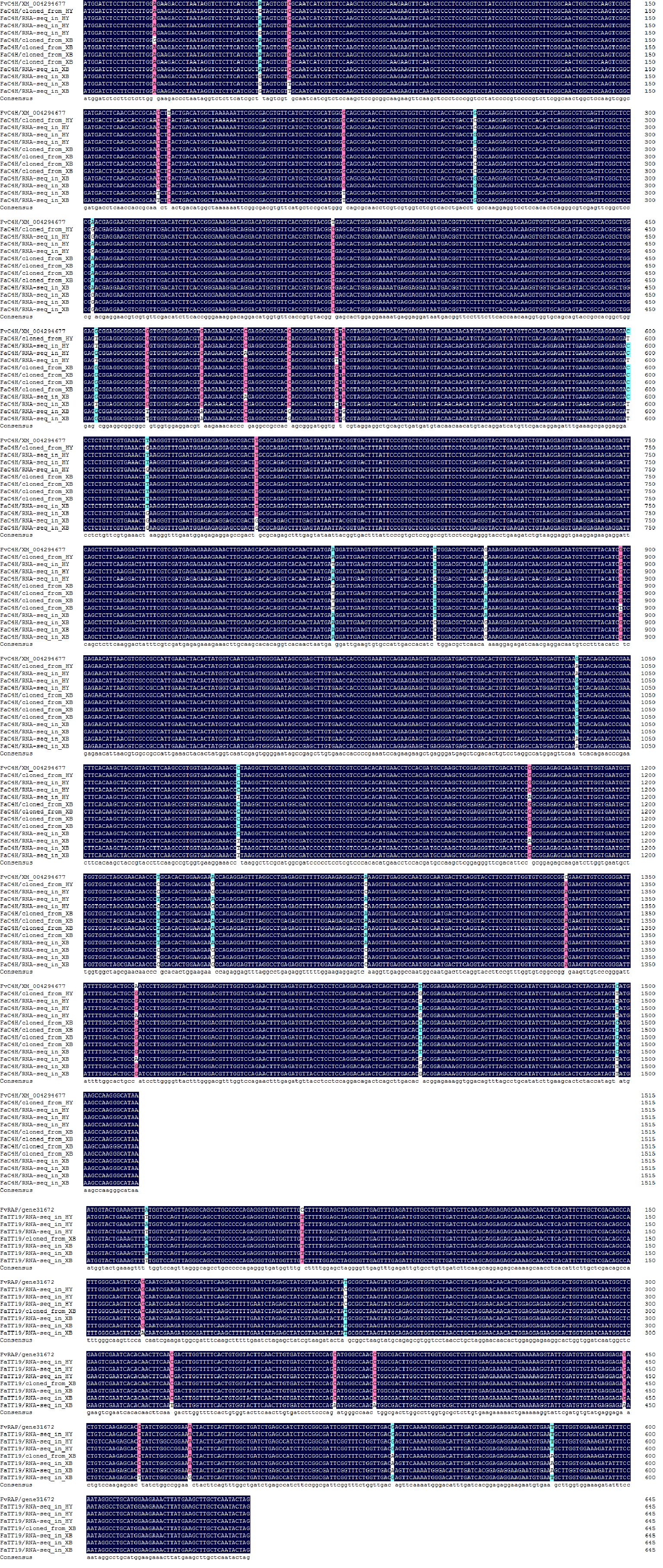

Supplement: Supplementary file 1 [file ijms-23-07375-s001.zip › Supplemental Figures/Figure S3.tif]

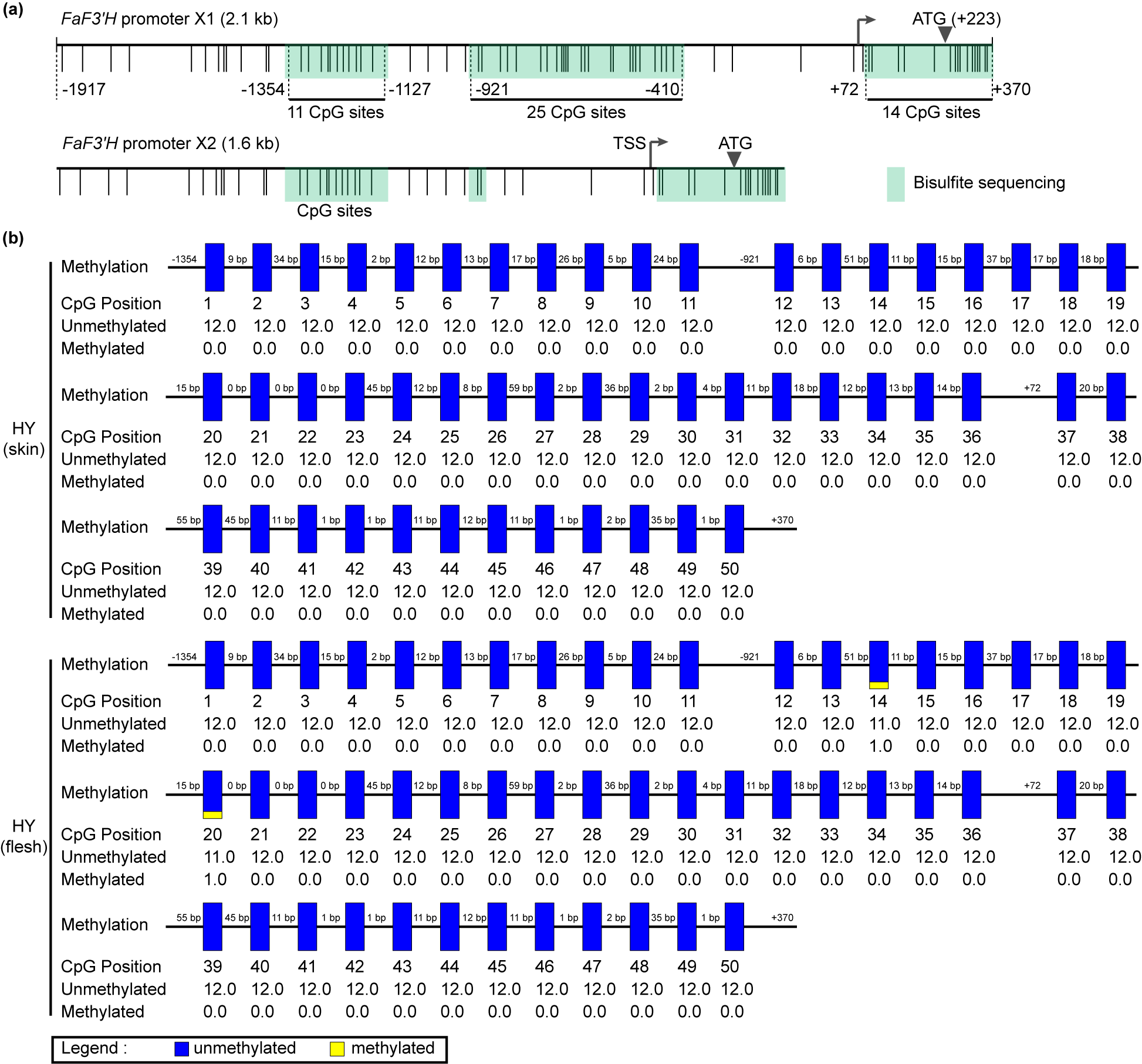

Supplement: Supplementary file 1 [file ijms-23-07375-s001.zip › Supplemental Figures/Figure S4.tif]

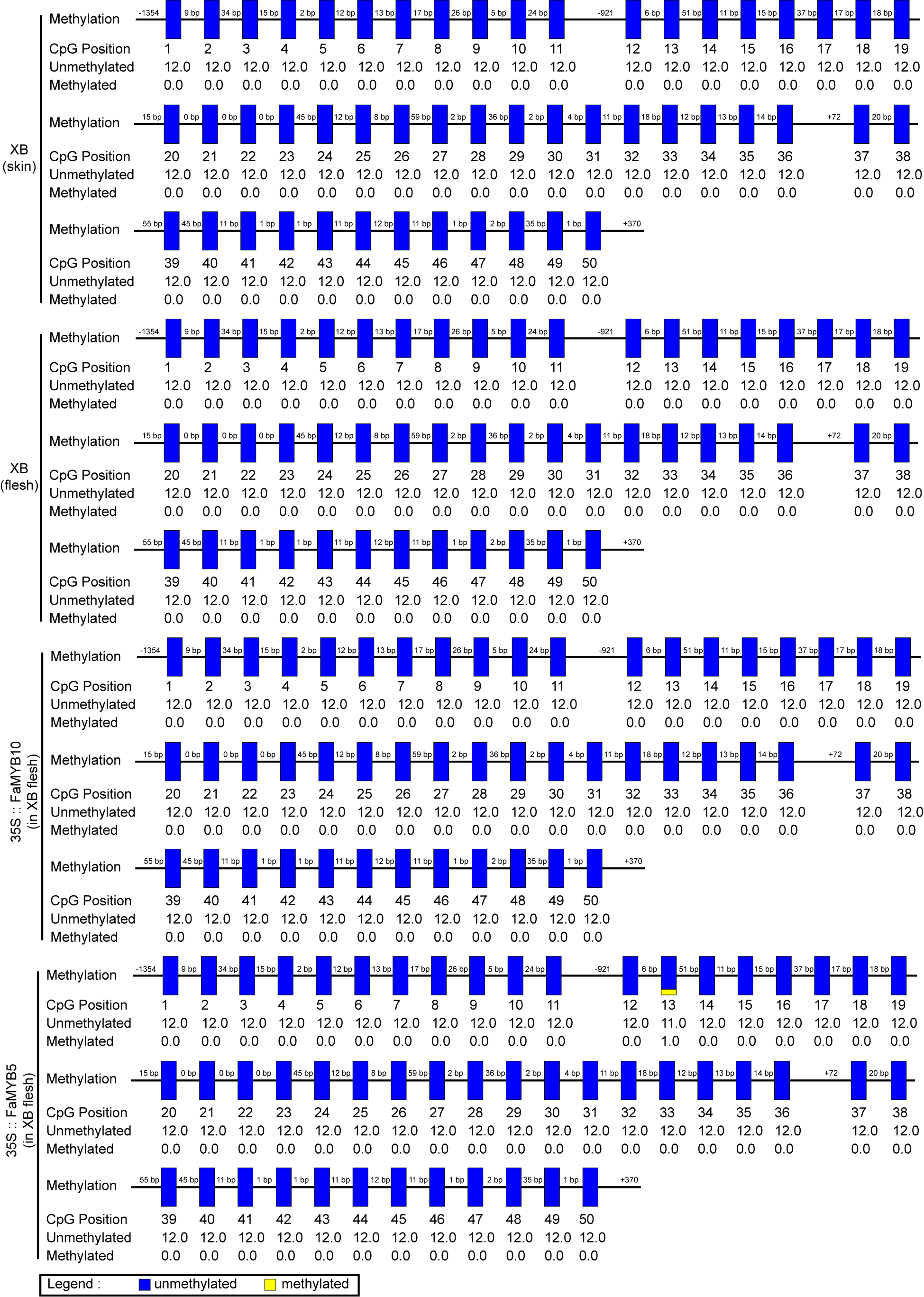

Supplement: Supplementary file 1 [file ijms-23-07375-s001.zip › Supplemental Figures/Figure S5.tif]
